# The prognostic significance of hematogones and CD34+ myeloblasts in bone marrow for adult B-cell lymphoblastic leukemia without minimal residual disease

**DOI:** 10.1038/s41598-019-56126-2

**Published:** 2019-12-23

**Authors:** Hongyan Liao, Qin Zheng, Yongmei Jin, Tashi Chozom, Ying Zhu, Li Liu, Nenggang Jiang

**Affiliations:** 1 Department of Laboratory Medicine, West China Hospital of Sichuan University, Sichuan, China; 2 Tibet Autonomous Region People’s Hospital, Lhasa, China

**Keywords:** Acute lymphocytic leukaemia, Prognostic markers

## Abstract

This study was aimed to dissect the prognostic significances of hematogones and CD34+ myeloblasts in bone marrow for adult B-cell acute lymphoblastic leukemia(ALL) without minimal residual disease(MRD) after the induction chemotherapy cycle. A total of 113 ALL patients who have received standardized chemotherapy cycle were analyzed. Cases that were not remission after induction chemotherapy or have received stem cell transplantation were excluded. Flow cytometry was used to quantify the levels of hematogones and CD34+ myeloblasts in bone marrow aspirations, and the patients were grouped according to the levels of these two precursor cell types. The long-term relapse-free survival(RFS) and recovery of peripheral blood cells of each group after induction chemotherapy were compared. The results indicated that, after induction chemotherapy, patients with hematogones ≥0.1% have a significantly longer remission period than patients with hematogones <0.1% (*p* = 0.001). Meanwhile, the level of hematogones was positively associated with the recovery of both hemoglobin and platelet in peripheral blood, while CD34+ myeloblasts level is irrelevant to the recovery of Hb and PLT in peripheral blood, level of hematogones and long-term prognosis. This study confirmed hematogones level after induction chemotherapy can be used as a prognostic factor for ALL without MRD. It is more applicable for evaluation prognosis than CD34+ myeloblasts.

## Introduction

B-cell acute lymphoblastic leukemia (ALL) is a common type of malignant tumor of the hematological system^1^. While the therapeutic effects are different among adult patients, chemotherapy is still the main treatment strategy in China^2,3^. The factors correlated with outcome include variable conditions of patients, such as age, drug resistance and treatment responses, and biological characteristics of leukemic cells, such as cytogenetic changes of neoplasms and the amount of leukemic cells^4–7^. Due to the crossing of multiple effects, it is difficult to predict the final prognosis according to a single factor. Although minimal residual disease (MRD) can be used as an independent prognostic factor for ALL^8–10^, the factor for further evaluating the long-term prognosis of MRD-negative cases is not yet available.

The precursor cells in bone marrow mainly include myeloblast and hematogones. Hematogones are the precursor cells of normal B lymphocytes, mainly recognized by flow cytometry immunophenotyping^11,12^. They can differentiate into functional mature B cells, so the level of hematogones in bone marrow is influenced by hematopoietic status and immune stimulation factors^13^. Hematogones in bone marrow can be more than 5% when hematopoietic function is activated after chemotherapy in neoplasm patients^12^. A few reports have indicated that the frequency of hematogones in bone marrow could be used as a prognosis indicator of AML, childhood ALL and B-cell ALL patients who have received hematopoietic stem cell transplantation, and suggested a better prognosis when hematogones >1% in childhood ALL^14–18^. However, it has not been thoroughly discussed whether the levels of hematogones and CD34+ myeloblasts in bone marrow have impact on the prognosis for adult ALL patients who have received chemotherapy. This study was focused on analyzing and comparing the relapse-free survival (RFS) of adult B-cell ALL patients with different levels of hematogones and CD34+ myeloblasts, and the correlation between precursor cells and recovery of peripheral blood cells was also evaluated.

## Results

### Characteristics of patients

A total of 113 adult patients with definitive diagnosis of B-cell ALL and received chemotherapy regularly in West China Hospital were selected, including 59 males and 54 females. Their ages ranged from 18 to 65 years and the median age was 37 years. The median follow-up time was 9.5 months. The detailed information was shown in Supplementary Table 1. All patients we selected have undergone courses of VDCLP (vincristine, daunomycin, cyclophosphamide, asparaginase, and dexamethasone) in induction chemotherapy, and 6-mercaptopurine (6-MP) and methotrexate (MTX) in maintenance therapy.

### Levels of hematogones and CD34+ myeloblasts in bone marrow

**Figure 1:**
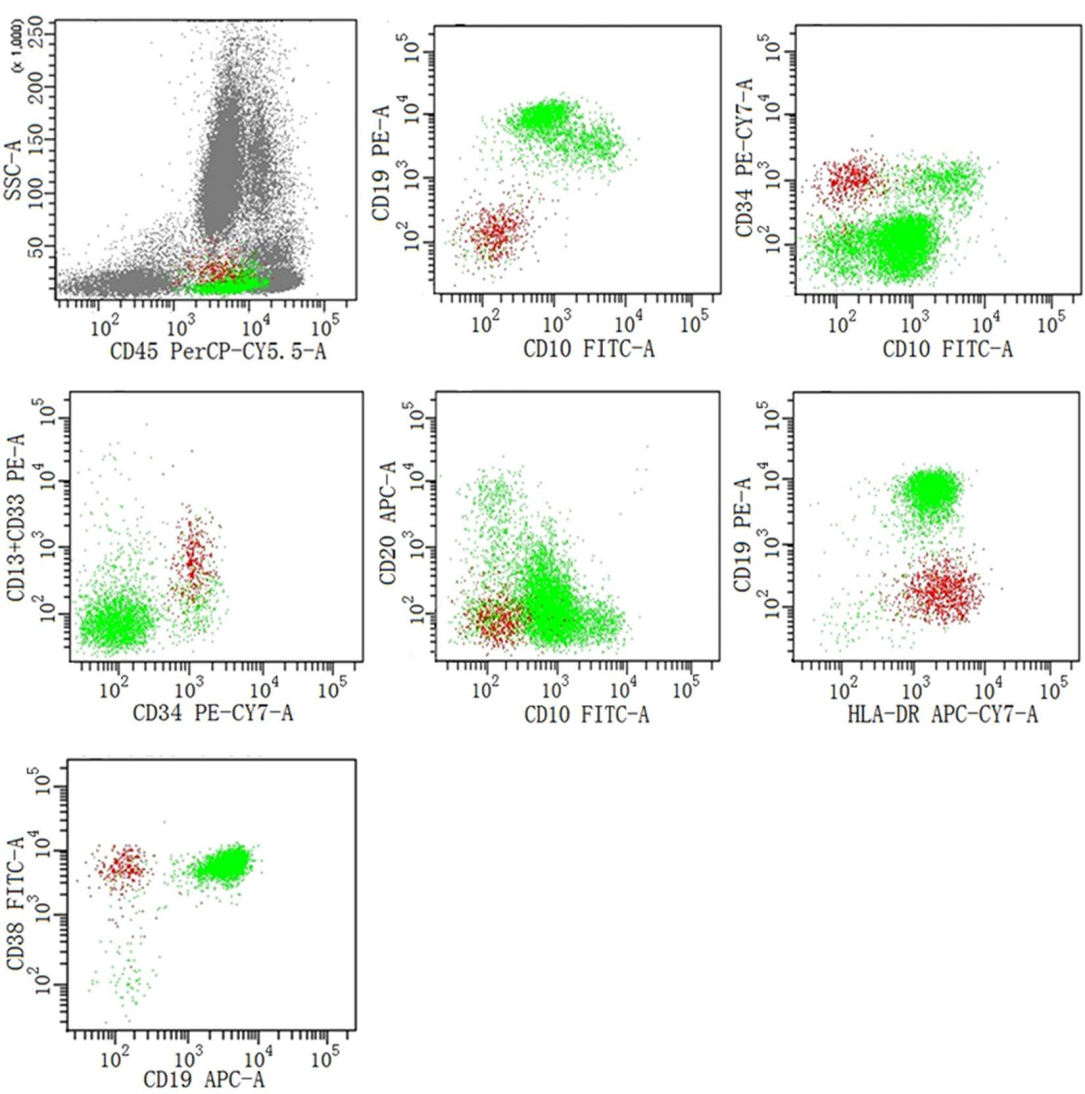
The identification of hematogones and CD34+ myeloblasts by flow cytometric immunophenotyping. On the dot plots, hematogones (green) expressed CD10 (partially), CD19, CD20 (partially), CD34 (partially), CD38 and HLA-DR, and CD34+ myeloblasts (red) expressed CD13/CD33, CD34 and CD38.

The identification of hematogones and CD34+ myeloblasts was achieved by flow cytometric immunophenotyping as indicated in Fig. 1. Although hematogones can be divided into different stages arbitrarily, we focused on the significance of the total level of hematogones in this study. Hematogones were detected positively in 78 cases only (69%), while CD34+ myeloblasts could be detected in all cases. In these adult patients, the levels of both hematogones and CD34+ myeloblasts were not correlated with age statistically (*p* = 0.214 and 0.234, respectively, Spearman correlation). Meanwhile, there was no correlation between the levels of these two types of precursor cells (*p* = 0.366). The percentage levels of hematogones and CD34+ myeloblasts in nucleated cells of bone marrow are listed in Table 1. These results indicated that the levels of hematogones were more variable than CD34+ myeloblasts among selected cases.

**Table 1:** The levels of hematogones and CD34+ myeloblasts in bone marrow.

	Hematogones	CD34+ myeloblasts
Range (%)	0–16.32	0.06–2.92
Median (%)	0.06	0.38
Mean ± SD (%)	0.97 ± 2.55	0.51 ± 0.52
CV	2.62	1.02

### RFS of adult B-cell all patients

**Figure 2:**
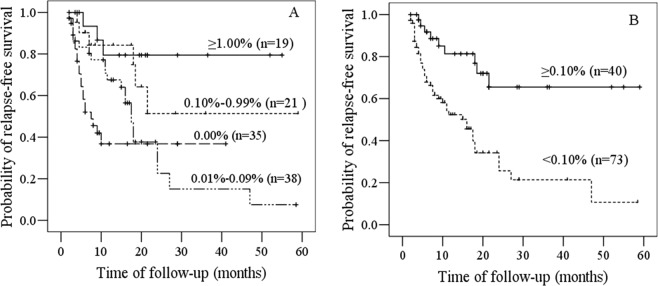
Kaplan-Meier survival curves of relapse-free survival according to levels of hematogones. (**A**) The RFS probabilities of 0.10–0.99% group and ≥1.00% group were higher than those of 0.00% group and 0.01–0.09% group. (**B**) The RFS probabilities of ≥0.10% group was higher than that of <0.10% group.

During the follow-up time, fifty one patients relapsed (45%), and the median time of RFS was 18 months. In order to define a reasonable cutoff value of the level of hematogones, we divided the cases into the following 4 groups according to the percentages of the hematogones in hematopoitic cells: 0.00% (35 cases), 0.01–0.09% (38 cases), 0.10–0.99% (21 cases) and ≥1.00% (19 cases). No significant difference was observed in RFS between the 0.00% group and the 0.01–0.09% group (*p* = 0.179), and between the 0.10–0.99% group and the ≥1.00% group (*p* = 0.305) (Fig. 2A). However, there were significant differences identified in the RFS of both the 0.00% group and 0.01–0.09% group comparing to the 0.10–0.99% group and ≥1.00% group respectively (*p* < 0.05) (Fig. 2A). Furthermore, we divided the cases into hematogones <0.10% group (73 cases) and hematogones ≥0.10% group (40 cases). Patients from the latter group showed better prognosis and there was a significant difference of RFS between these two groups (*p* = 0.001) (Fig. 2B).

**Figure 3:**
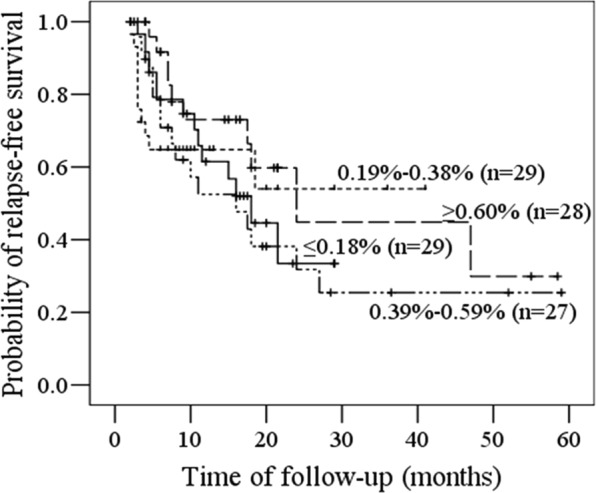
Kaplan-Meier survival curves of relapse-free survival according to CD34+ myeloblasts levels. The RFS probabilities of ≤0.18% group, 0.19–0.38% group, 0.39–0.59% group and >0.60% group were not significantly different.

Similarly, according to the percentage of CD34+ myeloblasts, we divided the cases into the following four groups: ≤0.18% (29 cases), 0.19–0.38% (29 cases), 0.39–0.59% (27 cases), and ≥0.60% (28 cases). As the levels of CD34+ myeloblasts were not variable as hematogones, the cases were grouped with equal percentiles. There was no significant difference in RFS among four groups (*p* = 0.536) (Fig. 3).

The multivariate Cox regression confirmed that white blood cells (WBC), age and hematogones grade were risk factors of prognosis (Table 2, Supplementary Table 1). Although it has been confirmed that absolute lymphocyte count(ALC) < 350 cells/μL predicts poor survival in several studies^19,20^, ALC in our study was not correlated with prognosis. This could be because the patients selected in our study were with better prognosis (gene markers and MRD negative) and the ALC of almost all cases was higher than 350 cells/μL, and the time point of blood cell count was different from previous study.

**Table 2:** Multivariate Cox regression in ALL.

	Odd ratio (95%CI)	*p*
Hematogones ≥0.10%	0.375 (0.175–0.805)	0.012
Age at diagnosis ≤25 y	0.419 (0.174–1.008)	0.052
Initial WBC ≥ 50,000cells/μL at diagnosis	2.029 (1.110–3.708)	0.021

### Comparison of peripheral blood recovery

Concentrations of hemoglobin (Hb) and platelet (PLT) were assayed at two time points and the cases were divided into groups according to the levels of hematogones or CD34+ myeloblasts as stated above. At the time of diagnosis, neither of Hb and PLT was significantly different between the hematogones <0.1% group and the hematogones ≥0.1% group (*p* = 0.384 and 0.847, respectively). One week after the completion of the induction chemotherapy, both Hb and PLT of hematogones ≥0.1% group were significantly higher than those of hematogones <0.1% group (*p* = 0.002 and 0.031, respectively), indicating that the hematopoietic recovery was faster in the group of hematogones ≥0.1% (Table 3).

**Table 3:** Hb and PLT concentrations (mean ± SD) in groups with different levels of hematogones at diagnosis and after chemotherapy.

		<0.1%	≥0.1%	*p* (T-TEST)
Hb (g/L)	At diagnosis	76.9 ± 25.5	83.8 ± 2.5.7	0.174
	After chemotherapy	84.4 ± 17.6	96.4 ± 18.6	0.001
PLT (×10^9^/L)	At diagnosis	73.6 ± 74.8	66.55 ± 52.0	0.581
	After chemotherapy	161.9 ± 67.9	191.7 ± 79.2	0.038

Hb: hemoglobin; PLT: platelet.

Among the CD34+ myeloblasts <0.18% group, 0.19–0.38% group, 0.39–0.59% group and ≥0.60% group, concentrations of both Hb and PLT in the peripheral blood were not different in statistics at diagnosis (*p* = 0.497 and 0.923, respectively). One week after the completion of induction chemotherapy, there was still no significant difference in Hb concentrations and PLT counts among these groups (*p* = 0.536 and 0.194, respectively), although these two blood parameters increased after chemotherapy in all groups (Table 4). This result indicated that the recovery degree of Hb and PLT was not correlated significantly with the levels of CD34+ myeloblasts.

**Table 4:** Hb and PLT concentrations (mean ± SD) in groups with different levels of CD34+ myeloblasts at diagnosis and after chemotherapy.

		≤0.18%	0.19–0.38%	0.39–0.59%	≥0.60%	*p* (ANOVA)
Hb (g/L)	At diagnosis	77.6 ± 24.9	76.8 ± 25.2	86.0 ± 28.5	77.3 ± 24.5	0.497
	After chemotherapy	91.3 ± 19.4	85.8 ± 17.1	91.1 ± 20.0	86.3 ± 18.7	0.536
PLT (×10^9^/L)	At diagnosis	72.8 ± 84.9	77.2 ± 83.1	68.4 ± 41.6.	65.5 ± 49.9	0.923
	After chemotherapy	168.8 ± 74.7	159.7 ± 56.2	163.8 ± 74.9	197.9 ± 82.5	0.194

Hb: hemoglobin; PLT: platelet.

## Discussion

Previous studies have indicated that precursor cells of both myeloid and lymphoid lines may arise accompany the reconstruction of bone marrow when leukemia achieves complete remission (CR) after induction chemotherapy^21,22^. In the present study, CD34+ myeloblasts can be detected in almost all adult B-cell ALL patients after therapy. However, hematogones were detected in part of the patients, and their percentages in nucleated cells were remarkably variable. While previous work showed that hematogones were more easily found in infants and adolescents^23^, our study focused on adult ALL patients, which removed the age factor that may significantly affect the level of hematogones.

Whether hematogones can be detected in the bone marrow is related to the hematopoietic microenvironment and immune factors^24–27^. For ALL patients, the proliferation of hematogones is inhibited by leukemic cells in bone marrow, and their growth is impaired by chemotherapy drugs. After leukemic cells are removed and chemotherapy drugs are eliminated, normal hematopoietic precursor cells may recover. The recovery level of hematopoiesis can be reflected by the parameters from a peripheral blood analysis. Our study indicated that there was no significant difference in Hb and PLT among the groups at the diagnosis of leukemia. However, after the completion of the induction chemotherapy, the hematopoietic recovery ability was better in patients with higher levels of hematogones. In fact, earlier reports have also demonstrated that hematogones were rarely detected in patients with myelodysplastic syndrome or aplastic anemia^28–31^. Therefore, our data consistent with previous work which suggest that the emergence of hematogones may be closely related to the active hematopoietic status.

In AML, hematogones have been identified as a prognostic factor^32,33^, and their appearance in bone marrow was also related to a lower incidence of graft-versus-host disease after stem cell transplantation^34^. However, the prognostic value of hematogones in adult B-cell ALL patients who have received chemotherapy remains poorly understood. The present study clearly demonstrated a positive correlation between the level of hematogones and the RFS of adult ALL cases. One possible explanation may be that the hematopoietic microenvironment is not conducive to the growth of normal hematopoietic cells in bone marrow without or with very few hematogones. Consequently, the leukemia stem cells are more likely to get growth advantage again during the long-term follow-up even in MRD-negative cases after inductive chemotherapy.

Previous studies have also shown an increased percentage of myeloid precursor cells during the recovery process of hematopoiesis^21,35^. Although only the subgroup of CD34+ myeloblasts was included in the current study, it represents the most important component of myeloid precursor cells. Our work suggested that the frequency of CD34+ myeloblasts had no correlation with Hb and PLT recovery and failed to predict a better prognosis for adult B-cell ALL patients. This may be due to the use of granulocyte colony-stimulating factors and secondary infections in some leukemia patients, both of which may stimulate the proliferation of myeloid progenitor cells^36–38^, and therefore compromise the rationality of applying CD34+ myeloblasts as an indicator of the intrinsic ability of hematopoietic recovery in bone marrow. In contrast, the frequency of hematogones in bone marrow is less affected by external factors, except autoimmune dysfunctions and a few viral infections^39–41^. In addition, our work revealed that the levels of CD34+ myeloblasts among the ALL cases were less variable than the percentages of hematogones, which made it more difficult to group the patients according to the CD34+ myeloblasts level and to define a cutoff value to evaluate the RFS statistically. Thus, we speculate that hematogones can reflect the hematopoiesis status of bone marrow more accurately and further serve as a better predicting factor for the long-term prognosis of B-cell ALL.

Our study definitively demonstrated that the level of hematogones in bone marrow after induction chemotherapy was correlated with hematopoiesis recovery and RFS. Furthermore, compared to CD34+ myeloblasts, hematogones could be used as a better prognosis factor for MRD-negative adult B-cell ALL patients. Considering the valuable insights that it may provide into clinical outcomes, the percentage of hematogones in hematopoietic cells should be reported simultaneously in MRD detection of B-cell ALL by flow cytometric immunophenotyping in clinical laboratories. Further risk stratifications of MRD-negative adult B-cell ALL according to hematogones levels may lead to treatment modifications or alternative treatment strategies in selective populations. Future research should figure out the different roles played by hematogones and CD34+ myeloblasts in the process of hematopoiesis. In addition, the prognosis value of hematogones need to be evaluated with wider spectrum of ALL cases, such as MRD-positive or with genetic abnormalities.

## Materials and Methods

### Patient recruitment and sample collection

From 2014 to 2018, the adult B-cell line ALL patients who were diagnosed and treated in West China Hospital of Sichuan University were enrolled in the current study. Bone marrow aspirations were extracted at the day 28 of induction chemotherapy and confirmed as MRD-negative by multi-parameter flow cytometry analysis. Genetic abnormalities are highly correlated with prognosis, such as *BCR-ABL1, TEL-AML1, KMT2A* translocations and gene markers related with *BCR-ABL1*-like ALL, and the treatments for these patients are also variable in our hospital. To guarantee the comparability among the cases and eliminating the influence of different genetic abnormalities and treatments variation on outcomes, only patients without specific recurrent genetic abnormalities (*BCR-ABL1*, *TEL-AML1, KMT2A* translocations and *BCR-ABL1-*like) who accessed chemotherapy and maintenance treatment regularly during the post-remission according to NCCN clinical practice guidelines and Chinese guidelines for diagnosis and treatment of ALL (2016)^42,43^ were enrolled, while patients who received stem cell transplantation were excluded. The gene markers are listed in Supplementary Table 2. In addition, to avoid the artificial reduction of precursor cell level in bone marrow caused by peripheral blood cell contamination, samples as follows were not taken into the study: (1) the total percentage of mature lymphocytes and monocytes >30%^44^; (2) Immature granulocyte and nucleated erythrocyte were not observed on bone marrow smears. This study was conducted in accordance with the Declaration of Helsinki, and the protocol was approved by the Ethics Committee of West China Hospital.

All patients were reviewed every 3 to 6 months and the time of relapse was recorded during the maintenance treatment period. The time of RFS was calculated in months. The criteria for loss of follow-up were: (1) not relapse during the observation period but lost to follow-up; (2) death from other diseases; (3) abandoning treatment.

### Detection of precursor cells

One microliter of bone marrow aspirations were extracted from the selected patients. Levels of hematogones and CD34+ myeloblasts were determined by a BD FACSCanto II flow cytometer (Becton Dickinson, US) and data was analyzed by the FACSDiva software. Antigen markers of CD10, CD19, CD20, CD34, CD38, HLA-DR, CD13, CD33 and CD45 (BD bioscience) were included for immunophenotyping (Table 5). After antigen labeling and cytometer detection, events with high side scatter (SSC) and low forward scatter (FSC) were excluded as signal interference on the FSC/SSC dot plot. Then the CD45dimCD19+ cells were gated and immunophenotyped to identify hematogones according to previous reports^45,46^, which was CD10+, CD34− or +, CD38+, CD20 + (partially), HLA-DR+, CD13− and CD33−.

**Table 5:** The antibody panels used for flow cytometric immunopenotyping.

	FITC	PE	PerCP-cy5.5	APC	PE-CY7	APC-CY7
Tube 1	CD10	CD19	CD45	CD20	CD34	HLA-DR
Tube 2	CD38	CD13 + CD33	CD45	CD19	CD34	

Myeloid progenitor cells are another substantial component of precursor cells in the bone marrow, but it is more difficult to define them distinctively as they are highly heterogeneous in derivation and immunophenotype. In this study, we only evaluated CD34+ myeloblasts with the immunophenotype of CD34+, CD13/CD33+, and CD19−.

The percentages of hematogones or CD34+ myeloblasts in nucleated cells were calculated and the correlation of these two precursor cells was analyzed. Then cases of ALL patients in the current study were divided into groups according to the levels of hematogones or CD34+ myeloblasts for subsequent analysis.

### Peripheral blood cell counting

Peripheral blood cells were counted by a Sysmex XN-9100 automated hematology analyzer at the time of B-cell ALL diagnosis and at day 7 after the completion of induction chemotherapy. To study the correlation between the level of precursor cells and the recovery of peripheral blood cells, the differences of Hb and PLT among the case groups with different levels of hematogones or CD34+ myeloblasts were analyzed statistically. The change of WBC were not used to evaluate hematopoietic recovery, as the quantity of which was significantly affected by invasive leukemic cells in peripheral blood during diagnosis.

### Statistical analysis

Spearman Rank Correlation was utilized to analyze the frequencies of hematogones and CD34+ myeloblasts. RFS among groups with different levels of precursor cells was plotted with Kaplan-Meier curves. Multivariate Cox regression was used to evaluate the potential risk factors. Comparison of peripheral blood cells among different groups was achieved by one-way ANOVA. A *p* value < 0.05 was considered to be statistically significant. The analyses were done with SPSS (version 20.0).

The datasets generated during the current study are available from the corresponding author on reasonable request.

### Ethical approval

All procedures performed in the study involving human participants were in accordance with the ethical standards of West China Hospital and with the 1964 Helsinki declaration and its later amendments.

### Informed consent

Informed consent was obtained from all patients for being included in the study.

## Supplementary information

Supplementary Table 1

Supplementary Table 2
