# Supplementary material for: The prognostic significance of hematogones and CD34+ myeloblasts in bone marrow for adult B-cell lymphoblastic leukemia without minimal residual disease
Source: Sci Rep. 2019 Dec 23;9:19722. doi: 10.1038/s41598-019-56126-2 (PMC6928064; doi:10.1038/s41598-019-56126-2)
Supplement: Supplementary file 1 — Supplementary Table 1 [file 41598_2019_56126_MOESM1_ESM.pdf]

The prognostic significance of hematogones and CD34+ myeloblasts in bone marrow for adult B-cell lymphoblastic leukemia without minimal residual disease

Hongyan Liao[1#], Qin Zheng[1#], Yongmei Jin[1], Tashi Chozom[2], Ying Zhu[1], Li Liu[1], Nenggang Jiang[1]*

[1]Department of Laboratory Medicine, West China Hospital of Sichuan University

[2]Tibet Autonomous Region People's Hospital

[#]These authors contributed equally to this work

*Corresponding author: Nenggang Jiang, M. D., West China Hospital of Sichuan University, Chengdu, China, Tel: 8618980606871, Email: 395066751@qq.com

Supplementary Table 1 Patients Information and Laboratory Results

Case NO.	Age (years)	Sex &	Hematogones percentage (%)	Hematogones grade #	Myeloblast percentage (%)	Myeloblast grade *	Follow-up time (month)	Outcome $	WBC(×10^9/L) at diagnosis	HGB(g/L) at diagnosis	PLT(×10^9/L) at diagnosis	HGB(g/L) after induction	PLT(×10^9/L) after induction	WBC(×10^9/L) after induction	Lymphocyte (×10^9/L) after induction
1	30	1	0.00	1	0.04	1	2	2	17.53	39	8	91	58	4.90	1.67
2	37	1	0.00	1	0.05	1	4.5	2	27.90	97	47	72	175	5.09	0.74
3	53	1	0.00	1	0.06	1	9	1	12.00	39	21	128	102	4.91	1.62
4	21	1	0.00	1	0.08	1	16.5	1	2.07	59	29	66	77	5.47	1.75
5	23	2	0.00	1	0.09	1	29	1	12.38	67	26	76	182	5.10	2.10
6	38	1	0.00	1	0.13	1	5.5	2	176.00	74	112	97	119	64.33	2.57
7	27	1	0.00	1	0.16	1	9.5	1	43.01	65	26	98	185	3.44	0.72
8	40	1	0.00	1	0.17	1	4	2	50.00	57	39	104	120	3.56	0.97
9	44	1	0.00	1	0.18	1	12	1	7.90	72	33	72	192	13.20	2.39
10	44	2	0.00	1	0.19	2	9.5	1	6.04	43	115	84	199	3.40	0.83
11	41	1	0.00	1	0.20	2	3	2	5.40	79	10	59	78	4.15	0.69
12	18	1	0.00	1	0.28	2	3	2	6.21	102	15	80	123	1.94	0.74
13	27	2	0.00	1	0.22	2	3.5	2	296.00	75	36	75	206	4.39	0.70
14	22	1	0.00	1	0.23	2	3	2	51.20	109	22	112	130	6.00	0.42
15	39	2	0.00	1	0.23	2	3	2	400.00	66	96	120	92	6.50	1.30
16	55	2	0.00	1	0.30	2	8	1	24.04	123	382	87	188	4.27	0.68
17	45	2	0.00	1	0.37	2	6	1	19.00	38	44	55	177	7.50	3.33
18	23	1	0.00	1	0.30	2	9	1	22.48	59	65	52	198	4.87	1.27
19	52	1	0.00	1	0.31	2	10	1	2.59	53	148	80	113	1.77	0.74
20	47	2	0.00	1	0.32	2	29	1	75.00	52	7	65	150	7.90	2.13

21	23	2	0.00	1	0.33	2	41	1	3.58	60	15	86	158	5.03	0.96
22	29	2	0.00	1	0.38	2	4	2	91.33	66	25	62	181	2.10	0.38
23	43	2	0.00	1	0.48	3	4.5	2	48.98	67	22	92	165	3.01	0.42
24	24	2	0.00	1	0.49	3	5	2	2.52	49	18	106	68	3.79	1.02
25	44	1	0.00	1	0.41	3	5	2	0.75	72	89	98	190	9.12	1.34
26	47	1	0.00	1	0.49	3	6	2	74.70	86	44	78	155	3.40	1.01
27	38	2	0.00	1	0.46	3	6	2	282.00	107	65	99	176	2.14	0.60
28	57	2	0.00	1	0.50	3	7.5	2	6.05	56	86	54	82	5.02	1.31
29	45	2	0.00	1	0.58	3	8	2	157.00	73	109	121	312	2.95	1.54
30	53	2	0.00	1	0.50	3	10	2	11.21	141	139	65	176	5.61	1.12
31	24	2	0.00	1	0.51	3	2	1	0.95	98	48	67	176	8.58	1.46
32	41	2	0.00	1	0.60	4	4	1	7.96	64	25	90	184	7.43	1.56
33	54	1	0.00	1	1.94	4	9	2	6.71	59	41	74	122	3.93	1.14
34	33	2	0.00	1	2.02	4	5.5	2	51.96	59	28	62	78	3.75	0.32
35	40	2	0.00	1	2.11	4	21.5	1	1.44	50	20	75	68	1.32	0.70
36	52	2	0.03	1	0.09	1	11.5	2	12.70	59	14	119	157	2.05	1.56
37	31	1	0.06	1	0.09	1	15	2	105.00	73	120	81	19	7.10	1.55
38	44	1	0.06	1	0.10	1	16	1	6.80	139	60	94	255	1.66	0.50
39	36	2	0.06	1	0.10	1	17	1	72.40	124	383	85	189	4.23	1.90
40	41	2	0.07	1	0.09	1	16	2	9.41	96	274	99	230	9.43	2.00
41	19	1	0.07	1	0.11	1	18.5	1	75.26	100	75	97	224	3.37	0.40
42	45	1	0.07	1	0.13	1	23.5	1	21.33	86	197	70	175	5.50	1.01
43	20	2	0.09	1	0.09	1	11	2	14.75	101	141	72	145	11.00	1.80
44	49	1	0.01	1	0.33	2	2	2	23.49	147	122	84	85	2.12	0.30
45	55	1	0.01	1	0.34	2	2.5	2	69.22	101	14	96	122	1.65	0.26
46	61	1	0.01	1	0.38	2	3	2	5.01	77	38	100	208	4.21	1.02

47	27	2	0.02	1	0.24	2	10.5	1	23.00	95	190	78	89	10.50	3.32
48	34	1	0.02	1	0.46	3	3	2	42.90	58	64	79	197	6.34	1.47
49	37	1	0.02	1	0.39	3	3.5	2	101.00	142	140	107	315	2.45	0.40
50	27	2	0.02	1	0.60	4	4.5	2	83.74	62	14	67	242	5.11	1.57
51	18	1	0.04	1	0.41	3	17.5	2	1.82	62	157	84	122	7.42	1.02
52	42	1	0.04	1	0.43	3	27	2	4.10	72	110	65	117	4.10	3.34
53	56	1	0.05	1	0.42	3	24	2	0.86	80	11	77	79	1.48	0.68
54	32	2	0.06	1	0.28	2	20	1	69.87	76	106	102	102	13.70	0.96
55	45	1	0.06	1	0.44	3	2	1	2.55	58	95	98	104	6.11	2.41
56	32	1	0.07	1	0.37	2	3.5	1	23.75	58	29	97	307	5.04	2.09
57	46	1	0.08	1	0.25	2	13	1	12.93	57	107	94	100	2.06	0.50
58	31	2	0.08	1	0.52	3	11	2	19.40	58	91	80	205	6.33	2.72
59	54	2	0.08	1	0.55	3	18	2	60.90	76	27	69	195	15.70	2.00
60	34	1	0.08	1	0.51	3	7	1	51.00	72	15	117	171	4.26	0.68
61	51	1	0.09	1	0.25	2	12.5	1	10.45	98	5	88	77	6.26	1.44
62	43	2	0.09	1	0.42	3	16	2	17.43	109	30	90	57	5.20	1.64
63	45	2	0.09	1	0.44	3	2.5	1	28.42	96	94	101	240	12.05	1.95
64	29	1	0.01	1	0.73	4	15	1	31.04	64	100	83	303	9.82	0.20
65	18	2	0.02	1	0.79	4	16.5	1	0.93	52	71	66	187	2.04	0.40
66	57	2	0.02	1	1.41	4	24	2	87.00	96	46	96	174	6.24	1.86
67	45	1	0.03	1	1.29	4	47	2	37.82	68	27	89	183	15.43	2.96
68	21	1	0.04	1	0.85	4	58.5	1	15.95	106	64	85	175	1.60	0.70
69	47	2	0.04	1	2.20	4	7	2	59.28	53	25	76	209	6.30	2.31
70	32	1	0.06	1	0.72	4	3	1	27.49	87	22	62	107	3.13	0.94
71	32	2	0.06	1	1.80	4	17.5	2	9.45	58	39	93	306	1.34	0.50
72	37	1	0.07	1	0.64	4	7.5	2	86.30	60	50	66	181	13.46	4.26

73	35	2	0.07	1	0.71	4	18	2	6.16	61	152	53	314	7.00	1.55
74	23	1	0.12	2	0.19	2	36	1	45.11	85	20	95	212	21.60	3.05
75	38	2	0.17	2	0.12	1	4	2	56.60	86	38	78	220	9.01	2.42
76	18	2	0.52	2	0.06	1	20	1	4.85	131	50	95	329	5.30	1.48
77	34	1	0.53	2	0.05	1	4	1	7.29	63	35	137	152	12.70	2.73
78	23	2	0.55	2	0.06	1	6	1	25.70	55	3	79	164	7.52	1.35
79	27	1	0.69	2	0.17	1	17.5	1	37.00	69	32	91	99	3.85	1.09
80	65	2	0.80	2	0.12	1	18	2	9.28	88	29	95	331	1.40	0.31
81	42	2	0.87	2	0.12	1	21.5	2	5.97	63	45	114	212	0.92	0.67
82	23	2	0.13	2	0.29	2	18.5	2	257.00	68	11	106	215	5.17	2.38
83	32	1	0.19	2	0.29	2	13	1	1.32	86	44	113	155	1.96	0.74
84	20	2	0.20	2	0.51	3	59	1	8.42	122	41	131	320	2.10	0.48
85	21	1	0.28	2	0.29	2	7	1	37.46	55	144	75	226	2.70	1.01
86	56	2	0.31	2	0.28	2	4.5	2	3.13	88	85	78	153	1.69	0.70
87	40	1	0.33	2	0.29	2	8.5	1	16.37	100	197	85	197	7.40	2.02
88	30	1	0.82	2	0.50	3	28.5	1	43.70	63	26	77	107	2.57	1.10
89	28	2	0.24	2	1.20	4	18	1	5.95	123	36	89	346	8.43	2.50
90	49	2	0.47	2	2.92	4	7.5	1	32.50	98	25	111	146	6.19	1.20
91	61	1	0.55	2	0.88	4	7	2	25.77	100	152	104	340	8.32	1.40
92	29	2	0.76	2	0.80	4	21.5	1	2.64	78	107	87	152	29.76	3.82
93	39	1	0.80	2	0.88	4	6	1	7.49	49	91	103	120	4.17	1.97
94	40	1	0.91	2	1.98	4	6	1	5.26	96	199	71	213	7.92	3.16
95	49	2	1.04	2	0.07	1	5.5	2	2.73	71	34	106	248	5.20	2.17
96	30	1	1.24	2	0.18	1	29	1	20.65	77	74	76	42	3.13	1.40
97	28	2	1.59	2	0.12	1	9	2	3.61	77	119	105	148	8.43	1.58
98	35	1	1.65	2	0.03	1	10.5	2	4.55	45	17	49	134	1.24	0.42

99	27	2	10.31	2	0.19	2	3.5	1	0.74	54	2	84	163	6.36	0.94
100	26	1	16.32	2	0.11	1	4.5	1	1.48	77	29	103	211	4.20	1.51
101	54	1	1.39	2	0.53	3	20	1	36.28	120	33	102	96	6.70	2.17
102	50	1	1.60	2	0.52	3	19.5	1	27.45	73	55	77	222	3.86	0.95
103	21	2	1.90	2	0.41	3	52	1	11.54	149	49	97	169	1.43	0.61
104	49	2	2.47	2	0.48	3	9	1	7.28	76	106	126	154	6.38	2.40
105	34	2	4.87	2	0.25	2	21.5	1	27.39	57	146	97	228	1.69	0.83
106	47	1	5.02	2	0.54	3	36.5	1	9.34	87	84	105	52	2.10	0.60
107	35	1	2.90	2	0.63	4	21.5	1	2.73	69	98	86	143	2.83	1.52
108	21	1	3.21	2	1.37	4	2	1	1.64	72	101	89	146	7.31	2.77
109	27	1	6.10	2	0.69	4	4	1	63.20	66	154	82	138	4.83	1.94
110	29	2	7.43	2	0.61	4	21	1	4.27	140	40	130	160	5.80	2.70
111	24	1	7.81	2	1.13	4	55	1	7.28	123	42	123	321	2.00	0.06
112	19	1	9.01	2	0.90	4	16	1	43.50	65	37	91	154	7.95	3.10
113	47	2	11.53	2	0.70	4	14.5	1	1.42	87	28	112	330	9.00	1.44

& 1: male, 2: female

1: hematogones <0.10%, 2: hematogones ≥0.10%

* 1: CD34+ myeloblast≤0.18%, 2: CD34+ myeloblast 0.19%-0.38%, 3: CD34+ myeloblast 0.39%-0.59% , 4: CD34+ myeloblast≥0.60%

$ 1: relapse-free, 2: relapsed
