# Supplementary material for: The prognostic significance of hematogones and CD34+ myeloblasts in bone marrow for adult B-cell lymphoblastic leukemia without minimal residual disease
Source: Sci Rep. 2019 Dec 23;9:19722. doi: 10.1038/s41598-019-56126-2 (PMC6928064; doi:10.1038/s41598-019-56126-2)
Supplement: Supplementary file 2 — Supplementary Table 2 [file 41598_2019_56126_MOESM2_ESM.pdf]

The prognostic significance of hematogones and CD34+ myeloblasts in bone marrow for adult B-cell lymphoblastic leukemia without minimal residual disease

Hongyan Liao[1#], Qin Zheng[1#], Yongmei Jin[1], Tashi Chozom[2], Ying Zhu[1], Li Liu[1], Nenggang Jiang[1]*

[1]Department of Laboratory Medicine, West China Hospital of Sichuan University

[2]Tibet Autonomous Region People's Hospital

[#]These authors contributed equally to this work

*Corresponding author: Nenggang Jiang, M. D., West China Hospital of Sichuan University, Chengdu, China, Tel: 8618980606871, Email: 395066751@qq.com

Supplementary Table 2 Gene Markers Detected in ALL patients

Common panel	BCR-ABL1-like related markers	
BCR-ABL1	ETV6-ABL1	SSBP2-CSF1R
TEL-AML1	ETV-JAK2	STRN3-JAK2
E2A-PBX1	PAG1-ABL2	RCSD1-ABL1
MLL-ENL	RSCD1-ABL2	PPFIBP1-JAK2
MLL-AF4	SNX2-ABL1	SSBP2-PDGFRB
MLL-AF9	ZMIZ1-ABL1	MYB-TYK
TLS-ERG	NUP214-ABL1	ETV6-NTRK3
CALM-AF10	ZC3HAV1-ABL2	ZEB2-PDGFRD
Hox11	TERF2-JAK2	MYH9-IL2RB
	PAX5-JAK2	EBF1-PDGFRB
	TPR-JAK2	EBF1-JAK2
	TNIP1-PDGFRB	RANBP2-ABL1
	ATF7IP-JAK2	CRLF2
	BCR-JAK2	IKZF3-8
	SSBP2-JAK2	
